# Physicians’ Practice of the Non-Cosmetic Uses of Botulinum Toxin: A Cross-Sectional Study in Saudi Arabia

**DOI:** 10.7759/cureus.21326

**Published:** 2022-01-17

**Authors:** Sarah A Alzarah, Huda Alabasi, Lujain Alanazi, Munirah Aldawsari, Etedal Aldawsari, Shazia Iqbal

**Affiliations:** 1 Internal Medicine, Vision Colleges, Riyadh, SAU; 2 Obstetrics and Gynecology, Vision Colleges, Riyadh, SAU

**Keywords:** physicians, non-cosmetic uses, btx, botox, botulinum toxoid

## Abstract

Background

Although botulinum toxin (BTX) has mainly been used cosmetically and therapeutically, its field of application is expanding. So far, BTX has shown promising outcomes in the management of a wide variety of medical conditions that are difficult to treat.

Objectives

We assessed physicians’ knowledge and experience regarding the non-cosmetic use of BTX in different clinical settings.

Methods

This is a cross-sectional survey that was conducted in Riyadh, Saudi Arabia, and included physicians from different specialties, with different levels of training, and from different working hospitals. Data were collected by using a self-administered survey to assess physicians’ knowledge, experience, current practice, and specific training in relation to Botox administration for non-cosmetic use.

Results

Most participants were residents (76.19%) and specialized in internal medicine (27.62%). The majority worked in governmental hospitals (76.19%). About 73% of our sample were aware of the non-cosmetic indications of BTX, but only 44% and 55% were aware of its contraindications and adverse effects, respectively. Less than one-third (31%) of respondents administered BTX injections in their clinical practice, and about 66% of respondents had two to five years of experience giving BTX injections. The most commonly treated conditions were spastic disorders, dystonia, and then migraine. Only 4.7% and 3.6% used validated scales or instruments for planning treatment with BTX, respectively, while about 36% opted for clinical evaluation only. More experience and training in giving BTX injections significantly predicted increased awareness of adverse effects and contraindications of non-cosmetic uses.

Conclusions

The majority of specialty groups in our sample were aware of the non-cosmetic applications of botulinum toxin but did not use them to the same extent in their practice. Additionally, only a minority wished for further education on injection practices. The finding of insufficient knowledge about contraindications and the adverse effects of injections in a large number of participants, however, highlights the need for increased education, especially given the wide range of non-cosmetic applications and benefits that BTX can have for a variety of diseases.

## Introduction

Botulinum toxin (BTX) is a protease exotoxin produced by a gram-positive, rod-shaped, anaerobic, spore-forming motile bacterium called Clostridium botulinum under anaerobic conditions [1]. This toxin inhibits the release of acetylcholine, a neurotransmitter responsible for the activation of muscle contraction and glandular secretion [2]. Injection of botulinum toxin type A has become one of the most popular cosmetic procedures worldwide. Botox is minimally invasive, can be administered relatively easily and quickly, and has an immediate and noticeable effect and a short recovery period [3].

Many botulinum toxin formulations are available commercially. However, the most commonly used formulations are abobotulinumtoxinA (BoNT-A), Dysport®, Ipsen Biopharm Ltd., UK; incobotulinumtoxinA, Xeomin®, Merz Pharmaceuticals GmbH, Germany; and onabotulinumtoxinA, Botox®, Allergan, Inc., USA [4]. Recently, botulinum toxin has been adopted by a growing number of physicians worldwide to manage a wide variety of medical conditions, including for the reduction of adductor laryngeal dystonia [5], as migraine prophylaxis [6], and for blepharospasm relief [4], rhinitis [7], hemifacial spasm, hyperhidrosis, achalasia [8], hand tremors [9], and urinary incontinence [10]. Moreover, it is used to manage various dental conditions such as temporomandibular joint disorders, bruxism, high lip line, and black triangles between teeth [4,11]. Research that investigates physicians’ knowledge, experience, practice, and education regarding the non-cosmetic use of BTX injection in clinical practice is still scarce, especially in the Middle East and developing countries. Therefore, we carried out this survey-based investigation in Saudi Arabia to assess the knowledge, experience, practice, and education of physicians in different specialties with regard to the use of BTX injections in clinical practice. This research will help in identifying any present gaps in the use of BTX injections for non-cosmetic purposes in order to maximize the future benefit of patients, as well as to improve physicians’ current level of knowledge.

## Materials and methods

Population and study design

This cross-sectional, self-administered questionnaire-based study was conducted among physicians of different specialties working in different hospitals in the city of Riyadh, Saudi Arabia, during the period from July to September 2021. A total of five hospitals were randomly selected from all hospitals and clinical settings in Riyadh City using a computer-generated randomization system. They included King Fahad Medical City, National Guard Hospital, Prince Mohammad Bin Abdulaziz Hospital, King Khalid University Hospital, and King Saud University Medical City. Physicians licensed by the Saudi Commission for Health Specialties (SCFHS) for different specialties were included. These specialties included neurology, neurosurgery, dermatology, urology, general surgery, otolaryngology, obstetrics and gynecology, internal medicine, ophthalmology, and dentistry. We excluded any licensed physicians in all specialties that are not authorized to use BTX injections in their clinical practice. Prior to conducting this research, ethical approval was obtained from the Second Health Cluster Institutional Board Institutional Review Boards (IRBs) Ethics Committee (Approval Number FWA00018774). Written consent was collected from all eligible individuals prior to participation.

Questionnaire structure

A self-administered questionnaire, which took around seven to 10 minutes to fill out, was completed by all eligible participants and is provided in the appendix. The questionnaire was adapted from a previously published paper after obtaining approval [12]. The questionnaire consisted of 17 items. The questionnaire was subdivided into five sections: (1) physicians baseline characteristics (specialty, level of training, and type of hospital they work in); (2) physicians’ knowledge and experience with BTX injections (indications, contraindications, and adverse effects of BTX injections); (3) physicians’ experience with BTX injections in clinical practice (current practice, presence of special unit in the hospital using BTX, years of experience, number of patients per year, conditions treated, commercial forms used); (4) physicians’ practice with the use of BTX injections (patient evaluations, recommended dosage, and reported cases of adverse effects); (5) physicians’ training in the use of BTX injections in clinical practice (certificates and training courses). The full questionnaire is provided in the Appendix.

Statistical analysis

Descriptive analyses were conducted using the Statistical Package for Social Sciences (SPSS, SPSS Inc., Chicago, IL). Statistical modeling was completed using R version 4.1.1 (R Core Team (2021)). For the logistic regression models, the dependent variables included awareness of adverse effects, contraindications, and non-cosmetic uses of BTX, and each was given a rating of either “aware” or “unaware.” One model was calculated for each of the three dependent variables. In each, independent variables included the area of specialty, level of medical training (either resident or specialist/consultant), having previous experience using BTX (either none or at least some), and having a certificate in BTX use. Interactions were not considered, as their inclusion in each model resulted in a worse Akaike Information Criterion (AIC), indicating that the benefits of their inclusion were outweighed by the loss of statistical power. In addition, while it was of interest whether a practice used BTX as a predictor of awareness, the use of BTX was highly collinear with the level of experience (r=0.86, p<0.001), resulting in an unacceptably high variance inflation factor (VIF=4.7 for each). As both could not be included, that which most improved the AIC in a model with the other predictors (specialty, level of training, certificate) was included. The current use of BTX was therefore excluded from the models.

## Results

Baseline physician characteristics

A total of 105 physicians were surveyed. The baselines for participants are presented in Table 1. The most common specialty of respondents was internal medicine (27.62%), followed by dentistry (23.81%). In terms of the level of training, residents constituted the majority of the study population, accounting for 76.19% of respondents while consultants were a minority, accounting for only 9.52%. The majority of physicians were working in the governmental sector (76.19%) while only a minority were working in the private sector (14.29%).

**Table 1:** Baseline physician characteristics

Variable	Category	Number (%)
Specialty		
	Medicine	29 (27.62%)
	Dentistry	25 (23.81%)
	Surgery	17 (16.19%)
	Obstetrics and gynecology	14 (13.33%)
	ENT and Ophthalmology	11 (10.48%)
	Pediatrics and family medicine	9 (8.57%)
Level of medical training		
	Resident	80 (76.19%)
	Specialist	15 (14.29%)
	Consultant	10 (9.52%)
Work sector		
	Governmental sector	80 (76.19%)
	Private sector	15 (14.29%)
	Other	10 (9.5%)

Physicians’ knowledge of BTX injections in clinical practice

The full details of physicians' knowledge of the applications of BTX injections in clinical practice can be found in Table 2. The majority of recruited physicians were aware of the non-cosmetic indications for the use of BTX injections in clinical practice (73.33%); however, less than half (44.76%) of respondents were aware of the possible contraindications for BTX injections. That being said, over half of our population were aware of the potential side effects associated with BTX injections (55.24%).

**Table 2:** Physicians’ knowledge of the applications of BTX injections in clinical practice (N = 105) BTX: botulinum toxin

Variable	Category	(%)
Do you know the non-cosmetic indications of BTX injection?		
	Yes	77 (73.33%)
	No	28 (26.67%)
Do you know the contraindications of BTX injection?		
	Yes	47 (44.76%)
	No	54 (51.43%)
	NA	4 (3.81%)
Do you know the adverse effects of BTX injection?		
	Yes	58 (55.24%)
	No	44 (41.90%)
	NA	3 (2.86%)

Physicians’ experience with BTX injection in clinical practice

This is described in detail in Table 3. Less than one-third (31.43%) of physicians used BTX injections in their clinical practice. Of note, 45.71% of physicians reported the presence of a particular specialized unit for the medical use of BTX injection in their hospitals and/or clinics. The majority of physicians (66.67%) reported no previous experience with the use of BTX injections for medical non-cosmetic uses. However, among those who used BTX injections in their clinical practice, most had two to five years of experience (11.43%), followed by two years of experience (9.52%). In terms of the medical reasons for the use of BTX injections, neuromuscular conditions were most frequently mentioned, specifically spasticity disorders (14.19%), migraine (12.90%), followed by dystonia (7.74%), dental applications (7.10%), and blepharospasm (6.45%). Other minor conditions were reported as well. Regarding the number of patients treated with BTX injections per year, the majority of respondents did not provide an answer (54.29%). However, among those who used BTX injections, the majority reported treating around 20 patients each year (5.71%), followed by 50 cases per year (2.86%), and 100 cases per year (1.90%). Among the available commercial formulations of BTX, onabotulinumtoxinA was the one most commonly used (12.61%), followed by abobotulinumtoxinA (13.51%), whereas botulinum toxin A was used the least (0.90%).

**Table 3:** Physicians’ experience with BTX injections' application in clinical practice BTX: botulinum toxin

Variable	Category	Number (%)
Do you currently use BTX in your clinical practice?		
	Yes	33 (31.43%)
	No	72 (68.57%)
Is there a specific unit in your hospital/clinic for the medical use of BTX injections?		
	Yes	48 (45.71%)
	No	57 (52.29%)
How many years of experience do you have with BTX injection?		
	>10 years	4 (3.81%)
	5-10 years	5 (4.76%)
	2-5 years	12 (11.43%)
	2 years	10 (9.52%)
	NA	4 (3.81%)
	None*	70 (66.67%)
What medical conditions do you treat with BTX injection?		
	Spasticity Disorders	22 (14.19%)
	Migraine	20 (12.90%)
	Dystonia	12 (7.74%)
	Dental	11 (7.10%)
	Blepharospasm	10 (6.45%)
	Overactive bladder	5 (3.23%)
	Hyperhidrosis	4 (2.58%)
	None	37 (23.87%)
	Miscellaneous	22 (14.19%)
	NA	11 (7.10%)
How many patients (per year) do you treat with BTX injections?		
	0 -10	34 (32.3%)
	11-20	7 (6.66%)
	21-30	1 (0.95%)
	30-40	1 (0.95%)
	50-60	3 (2.86%)
	90-100	2 (1.90%)
	NA	57 (54.29%)
Which commercial form of BTX injection do you use?		
	Abobotulinumtoxin A	15 (14.28%)
	Incobotulinumtoxin A	6 (5.71%)
	Onabotulinumtoxin A	14 (13.3%)
	None	62 (59 %)
	NA	14 (13.3%)

Physicians’ practice regarding the medical use of BTX injections

Physicians’ injection of BTX in clinical practice is summarized in Table 4. The majority of physicians based their clinical preference for using BTX injections of different formulations on clinical evaluation (36.04%). In terms of the frequency of BTX re-injection, most respondents did not provide an answer (60%) and 17 respondents (16.19%) reported no re-injections at all. Around one-third of physicians, especially those who use BTX injection in their practice, reported using BTX injections in the recommended dilutions (28.57%) and 32.38% of physicians found the recommended dose of BTX injection to be adequate. That being said, a minority of physicians (3.81%) reported adverse events associated with the medical use of BTX injections.

**Table 4:** Physicians’ use of BTX injections BTX: botulinum toxin

Variable	Category	(%)
How do you evaluate patients in order to plan a treatment strategy based on injection of BTX?		
	Clinical evaluation	40 (36.04%)
	Instrumental evaluation	4 (3.60%)
	Validated scales	5 (4.50%)
	NA	62 (55.86%)
Do you inject BTX according to the recommended dilutions?		
	Yes	30 (28.57%)
	No	11 (10.48%)
	NA	64 (60.95%)
Have you ever reported at least one adverse reaction to BTX in your clinical practice?		
	Yes	4 (3.81%)
	No	34 (32.38%)
	NA	67 (63.81%)

Physicians’ education and training in the use of BTX injections

Data regarding physicians’ education and training in the non-cosmetic use of BTX injections are fully presented in Table 5. Only a minority of participating physicians have specific certification for the practice of BTX injections in clinical practice, which accounts for 9.52% of all respondents. A limited number of physicians reported taking training and courses related to BTX application in practice: 5.71%, 5.71%, and 0.95% of physicians took one, two, and five courses, respectively.

**Table 5:** Physicians’ training in the use of BTX injections in clinical practice BTX: botulinum toxin

Variable	Category	(%)
Do you have a specific certificate to practice BTX injection?		
	Yes	10 (9.52%)
	No	92 (87.62%)
	NA	3 (2.86%)
How many courses on attended in the 2 past years?		
	0	29 (27.62%)
	1	6 (5.71%)
	2	6 (5.71%)
	5	1 (0.95%)
	NA	63 (60.00%)

Effect of the area of specialty, level of training, and previous BTX injection experience

**Figure 1:**
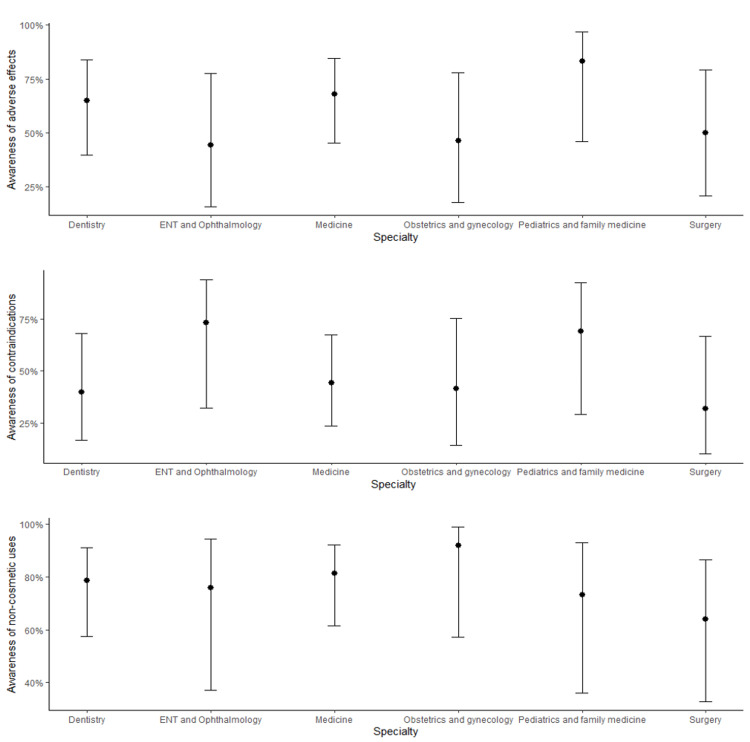
Awareness of adverse effects, contraindications, and non-cosmetic uses by area of specialty

**Figure 2:**
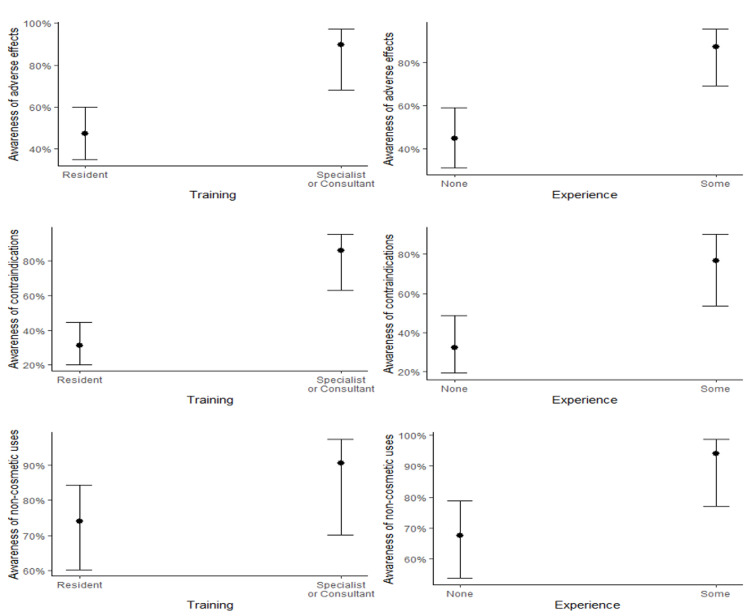
Awareness of adverse effects, contraindications, and non-cosmetic uses by the level of training and experience

Logistic regression modeling showed acceptable fit for awareness of adverse effects (AUC=0.792, sensitivity=0.727, specificity=0.767, p<0.001), contraindications (AUC=0.843, sensitivity=0.864, specificity=0.746, p<0.001), and non-cosmetic uses (AUC=0.745, sensitivity=0.608, specificity=0.815, p=0.007). Multicollinearity was negligible (All VIF < 1.07). The significance of effects of area of specialty, level of training, and level of experience on each of the awareness of adverse effects, contraindications, and non-cosmetic uses are outlined in Table 6. Briefly, while there was variability in these outcomes across areas of specialty (Figure 1), these differences were not significant. However, having had experience with BTX injection was significantly associated with higher awareness in all three categories, and level of training was significantly associated with higher awareness of adverse effects and contraindications (Figure 2).

**Table 6:** Significance of all variable effects in regression modeling Indicates significance at a level of 𝛼 = 0.05*

Model	Effect	Significance
Awareness of adverse effects	Area of specialty	F(5,90)=0.72, p=0.611
	Training	β=2.28, SE=0.77; F(1,90)=11.05, p=0.001 *
	Experience	β=2.17, SE=0.67; F(1,90)=11.93, p<0.001 *
Awareness of contraindications	Area of specialty	F(5,89)=0.78, p=0.568
	Training	β=2.61, SE=0.72; F(1,89)=16.63, p<0.001 *
	Experience	β=1.92, SE=0.67; F(1,89)=8.87, p=0.004 *
Awareness of non-cosmetic uses	Area of specialty	F(5,93)=0.57, p=0.728
	Training	β=1.21, SE=0.76; F(1,93)=2.67, p=0.105
	Experience	β=2.01, SE=0.84; F(1,93)=7.07, p=0.009 *

## Discussion

This study assessed the knowledge of the non-cosmetic use of BTX injections across different medical specialties. Recently, the usage of botulinum toxin injection (Botox) has grown because of its effectiveness, acceptability, and minimally invasive procedure. Worldwide, botulinum toxin injections are used for both cosmetic and non-cosmetic indications. They have shown promising outcomes in the management of different medical conditions or specific patients that seemed unresponsive to commonly used therapies (for example, see [13-15]). Most of the samples do not routinely use Botox in their practice (68%), but those who do use it mostly for spastic disorders, migraine, and dystonia. Similar findings are observed in other research, where it is evident that Botox is employed to treat a wide range of non-cosmetic illnesses such as spasmodic dysphonia, voice tremor, masticatory myalgia, headache, and cervical dystonia [12]. Among the biochemical formula, abobotulinumtoxinA and onabotulinumtoxinA are the preferred choices in the region. Such findings are consistent with results in an already published article, which noted that abobotulinumtoxinA and onabotulinumtoxinA are the drugs of choice for head and neck indications [16].

The study determined that most of the practitioners had no experience of using botulinum toxin injections. This was evident from the finding that only 31.43% of participants had treated patients with BTX injections over the last year, and 66.67% had no experience at all with Botox. In our study, there was reasonable awareness of the use of BTX injections for non-cosmetic indications: about 73.33% were aware of such indications while only 44.76% and 55.24% were aware of Botox contraindications and adverse effects, respectively. This might be explained by the lack of experience reported by many respondents and the fact that the majority of our sample consisted of residents (76.19%) who might not be allowed to perform such injections at this point in their training.

Among those that do treat patients with BTX injections, we found that 10.4% of participants do not adhere to the recommended dosages of BTX. This might be explained by experienced practitioners adapting the dosage to the size (increasing dosage with bigger size) and/or to the location of the targeted muscle (e.g., lower dosage when injecting close to a motor endplate) [17]. Because Botox may trigger dangerous adverse events. which have been observed sometimes with therapeutic use, as well as observed after minor cosmetic procedures [18], practicing physicians should adhere to current standards of practice and standard dosage, which are crucial to avoiding side effects and complications rate [18]. Luckily, less than 4% of our sample reported adverse effects after Botox injection. Furthermore, in our sample, only 36.04% of practicing physicians reported using only clinical evaluation to decide upon Botox injection. Previous studies documented that the use of visual guidance techniques, or even no use of such guidance techniques at all, is less precise, requires higher dosages, and is overall suboptimal [13]. They identified the barriers to the use of guidance techniques such as electromyography (EMG) to be the need for equipment and training [13].

Demand for structured training programs regarding the non-cosmetic use of botulinum toxin

We were able to show that more experience and training with BTX injections, in particular, were significantly related to higher awareness in all three areas, including the use of non-cosmetic applications, adverse effects, and contraindications of the non-cosmetic applications of Botox. That said, we also found that only 9.52% of physicians held a specific Botox training certificate for this treatment. The history of BTX shows that its therapeutic use covers a wide range and has been constantly evolving since its discovery [19]. The increase in non-cosmetic applications of BTX is further exemplified by a recent survey of physicians, in which 86% of respondents using BTX to treat cervical dystonia and spastic paresis reported a significant increase in patients treated with BTX injections over the last five years [20]. For professions that treat these two conditions with BTX, Ipsen Biopharm (Wrexham, United Kingdom) created its Ixcellence Network® in 2012 to educate practitioners on best practices and to standardize the treatment methods [21]. This exemplifies that, to expand the medical, non-cosmetic use of botulinum toxin, practicing physicians benefit from training and proper knowledge of indications; adverse effects or contraindications; as well as practice guidelines such as injection intervals and dosages [22]. It has been established that BTX injections significantly improved the quality of life of stroke patients suffering from spasticity [23]. However, the lack of effectiveness could be linked to many reasons, including inadequate dosage, imprecise injection site selection, and limited knowledge regarding injection techniques [24]. It has been reported in previous literature that delivery of injections without instrumental guidance was practiced in 25% of post-stroke spasticity cases [25]. Most importantly, late management of a significant number of post-stroke spasticity cases has been reported, which increases the likelihood of severe muscle hypertonia and contracture, emphasizing the importance of early planning of treatment for stroke survivors [26]. On the other hand, BTX injections have shown long-term prophylaxis of chronic migraine with good tolerability and improvement of quality of life in severe persistent cases by decreasing the incidence of depression and enhancing work productivity [27-28]. In some cases, this can only be achieved by multiple cycles of BTX injections supported by the fact that 33% of chronic migraine patients who did not respond to the first cycle showed good or partial response during the second one [29], whereas the proportion of patients who responded well to BTX injections are those with shorter chronic migraine duration and fewer headache days [30]. All of which points to the importance of better training and knowledge of the therapeutic uses of BTX injections for better practice. Organizing instructional programs and short courses regarding non-cosmetic indications and contraindications can ensure the safety of botulinum toxin use among general practitioners. Experienced physicians can be trained to enhance the effectiveness of injections, and such training might cover 1) ongoing assessment using validated scales and instruments, 2) choice of the injection site, 3) using tools like ultrasounds for guided injections, and 4) choice and proper implementation of rehabilitation plans (see [20]). Training like this might not only elevate the specific knowledge about and the effectiveness of BTX injections but additionally increase confidence in the practitioners in a healthy way, improving treatment even further [20].

Limitations

We did not directly assess what kinds of training participants followed - certified or not - that made them eligible to safely inject BTX solutions. In a similar survey, 98% of the participants received some training before their first injection, with 77% being trained by a colleague, 65% taking part in practical sessions, and 65% attending theoretical courses [20]. While some only had one of these learning opportunities, 42% of the participants in that survey received all three training types. It would have been interesting to see if there was a similar lack of standardization in the types of training our participants underwent. It was, unfortunately, not possible to include the answers on certificates in the statistical analyses due to the high homogeneity of data connected to this question. Additionally, the majority of our sample consisted of residents. Therefore, their training is most likely incomplete, and we found their level of education to explain the high number of participants without proper knowledge about adverse effects and contraindications. These participants might also have biases, for example, the years of education reported and the number of certificates for BTX injections. A more diverse sample, not just targeting different specialties but also looking for more equal numbers of residents vs. specialists and other variables connected to the level of training, should therefore be pursued in future studies. Looking at the results of a study pursuing a similar goal to ours [12], our survey could have, additionally, been extended to include questions about the guiding techniques used by the practitioners during injection, the side effect of the injections, and how they care for their patients afterward (i.e., prescription of rehabilitation treatments like electrical stimulation or physical therapy). This information might have helped establish a more detailed picture of the physicians' knowledge of the non-cosmetic use of BTX injections.

## Conclusions

To our knowledge, this is the first survey assessing non-cosmetic BTX training and injection practices across this broad spectrum of professions and target diseases in Saudi Arabia. It serves to compare the levels of knowledge across professions and determine what kind of BTX injection training, in general, is wanted and needed. Lack of awareness about contraindications and/or the adverse effects of the non-cosmetic use of Botox among our participants provokes a high demand for mandated formal training programs and authentic certificates in order to ensure the safe use of Botox by general practitioners and clinicians. Pilot studies assessing the effectiveness of different training methods for specific non-cosmetic applications of BTX are a possible next step, hopefully enabling easily accessible and effective training programs in the near future.

## Appendices

Description of the self-administered questionnaire assessing physicians’ knowledge and experience, practice, education/training for the medical non-cosmetic use of botulinum toxin injection in clinical practice

Study Title: Physicians’ Practice of Non-cosmetic Uses of Botulinum Toxin: A Cross-sectional Survey

“The research is aiming toward the knowledge of using botulinum toxins in non-cosmetics ways regardless if you use it or not in your specialty, so kindly participate in filling this survey“

The purpose of the study is to understand practice status and the experience of physicians in using botulinum toxin injection for treating various medial disorders in Riyadh, Saudi Arabia. You are eligible to participate because assessment of physicians’ practice in this field would be helpful for improving the clinical practice and patient’s benefit. If you agree to participate, your participation will involve completing a survey. It should take no more than 10 minutes. You may choose not to answer some or all of the questions. Your name will not appear on your completed survey, and no identifying information is being collected as part of this survey.”

Personal data:

1. Participant’s Name (optional): ……….………………….  

 2. ID (optional): …………………………...……………………

3. * Workplace: ……………………………………………….

**Table 7:** Description of the self-administered questionnaire assessing physicians’ knowledge and experience, practice, and education/training for the medical non-cosmetic use of botulinum toxin injection in clinical practice

Item	Questions	Answer
Specialty, Degree and work place		
1	What is your specialty?	……………..
2	What is your medical degree?	……………..
3	Are you working at government or a private hospital?	Governmental sector
		Private sector
		others
Knowledge and Experience		
1	Do you know the non-cosmetic indications of botulinum toxin injection?	Yes
		No
2	Do you know the contra-indications of botulinum toxin injection?	Yes
		No
3	Do you know the adverse effects of botulinum toxin injection?	Yes
		No
4	Do you currently use botulinum toxin injection in your clinical practice?	Yes
		No
5	Is there a specific service for treating patients with botulinum toxin injection in your hospital/ center/ unit?	Yes
		No
6	How many years of experience do you have with BTX injection?	10 Years
		5-10 Years
		2-5 Years
		2 Years
		None
7	What medical conditions do you treat with botulinum toxin injection?	Spasticity Disorder
		Dystonia
		Blepharospasm
		Migraine
		Dental
		None
		other
8	How many patients/year do you treat with botulinum toxin injection?	
9	Which commercial formulation of botulinum toxin you usually use?	AbobotulinumtoxinA
		IncobotulinumtoxinA
		OnabotulinumtoxinA
		None
		Other
Practice		
1	How do you evaluate patients in order to plan a treatment strategy based on the injection of botulinum toxin?	Clinical evaluation
		Instrumental evaluation
		Validated Scale
		Not applicable
2	Do you inject botulinum toxin according to the recommended dilutions?	Yes
		No
		Not applicable
3	Have you ever reported at least one adverse reaction to botulinum toxin in your clinical practice?	Yes
		No
		Specify
		Not applicable
Education/training		
1	Do you have a certificate to practice botulinum toxin injection?	Yes
		No
2	How many courses on attended in the 2 past years?	Number……………
